# Effect of Strain on the Electronic Structure and Phonon Stability of SrBaSn Half Heusler Alloy

**DOI:** 10.3390/molecules27123785

**Published:** 2022-06-12

**Authors:** Shakeel Ahmad Khandy, Ishtihadah Islam, Kulwinder Kaur, Atif Mossad Ali, Alaa F. Abd El-Rehim

**Affiliations:** 1 ZJU-Hangzhou Global Scientific and Technological Innovation Center, School of Micro-Nano Electronics, Zhejiang University, Hangzhou 311200, China; 2 Department of Physics, Jamia Millia Islamia New Delhi, New Delhi 110025, India; ishtihadahislam@jmi.edu.in; 3 Department of Applied Sciences (Physics), Punjab Engineering College (Deemed to Be University), Chandigarh 160012, India; kulwinderkaur@pec.edu.in; 4 Department of Physics, Faculty of Science, King Khalid University, Abha 61413, Saudi Arabia; atifali@kku.edu.sa (A.M.A.); alaa.abdelrehim@kku.edu.sa (A.F.A.E.-R.); 5 Department of Physics, Faculty of Science, Assiut University, Assiut 71516, Egypt; 6 Department of Physics, Faculty of Education, Ain Shams University, Roxy, Cairo 11771, Egypt

**Keywords:** electronic structure, half Heusler alloys, phonon properties, elastic constants

## Abstract

This paper presents the strain effects on the structural, electronic and phonon properties of a newly proposed SrBaSn half Heusler compound. Since it is stable considering chemical thermodynamics, we tested its strength against uniform strain w.r.t phonon spectrum and it produces a direct bandgap of 0.7 eV. The direct bandgap reduces to 0.19 eV at −12% strain beyond which the structure is unstable. However, an indirect gap of 0.63 eV to 0.39 eV is observed in the range of +5% to +8% strain and afterwards the strain application destabilizes the structure. From elastic parameters, the ductile nature of this material is observed.

## 1. Introduction

The search for technologically useful functional and physically interesting materials has recently pushed plentiful suggestions of proposed materials with stimulating properties assured by first-principle calculations. In this regard, electronic structure theory has been extended to propose novel compounds in presumed structural configurations, in search of useful functional materials [1,2,3]. Zhang et al. within this domain reported 235 compounds to be thermodynamically and electronic structures of 18 additional materials were investigated, seeking potential new material functionalities [4]. Half Heusler (HH) compounds, since their discovery, have expanded to a database of several hundred due to their phenomenal applications such as spin polarization [5,6,7], thermopower [3,8,9,10,11,12,13,14], (anti-ferro/ferro/ferri) magnetism [15,16,17], superconductivity [18,19] and topological effects [2,19,20,21]. After shape memory and spintronics, half Heusler compounds are mainly investigated or re-discovered for intriguing thermoelectric properties as some of these materials display larger seebeck and electrical conductivity values [22]. A high ZT of 0.7–1.5 in MNiSn (M = Ti, Zr, Hf) has been reported [9]. Sakurada et al. from first-principle calculations predicted a ZT of 2.68 for KBiBa. Similarly, FeNbSb-based materials always present a ZT value greater than unity [12,23]. While investigating XIrSb (X = Ti, Zr, Hf) alloys by density functional theory methods, figure of merit in p-type doping of XCoSb (X = Ti, Zr, Hf) compounds premediated a ZT value of 1.0 at 1097 K [11,24]. TrIrSb is found to display a maximum ZT = 0.95 at 800 K [8]. In our previous studies, we explored the thermoelectric and phonon properties of PdTaX (X = Al, Ga, In) materials from first-principle calculations with smaller thermal conductivity values [25]. Among quaternary Heuslers, CuLiX(X = Se, Te) are believed to be decent thermoelectrics with a high ZT value of 2.65 for CuLiTe and 1.7 for CuLiSe [26]. RuTaSb by Fang et al. [27] was reported to display large band degeneracy accompanied by low effective mass, which directs its ZT to elevate up to 1.5 at 1200 K. Inspired from the above-stated discussion, we tried to investigate the newly reported SrBaSn half Heusler alloy [4] using DFT simulations. In the previous report, only the structural details alongside bandgap are elaborated and thus we explored electronic properties, strain engineering as well as its stability concerns in detail (phonon, chemical, mechanical) in this manuscript.

## 2. Computational Details

All the DFT-based calculations are executed within the full potential linear augmented plane wave (FP-LAPW) method employed in WIEN2k code. We have applied generalized gradient approximation (GGA) with Perdew Burke and Ernzerhof (PBE) [28] for exchange-correlation to optimize and relax the system and for electronic structure and transport calculations, we make use of the advanced TB-mBJ method [29]. We have utilized 10 × 10 × 10 *k* points and the tetrahedral method for the Brillouin zones integration under the Monkhorst-pack scheme [30]. The charge and total energy are converged by 10^−4^ e and 10^−4^ Ry, respectively. Phonon calculations are estimated using Quantum Espresso code [31] with (PBE-GGA) approximation. Norm-conserving pseudopotential under cutoff 60 Ry for kinetic energy and 600 Ry for charge density are employed. To achieve an agreement for both Wien2k and Quantum Espresso calculations, we checked the ground state energy and k-point convergence. We applied a uniform expansive and compressive strain from −15% to +10% of a_o_ in both directions. From phonon calculations, we restricted ourselves to positive frequencies only and hence further calculations were carried out on the stable phases accordingly.

## 3. Results and Discussion

### 3.1. Structure

**Figure 1:**
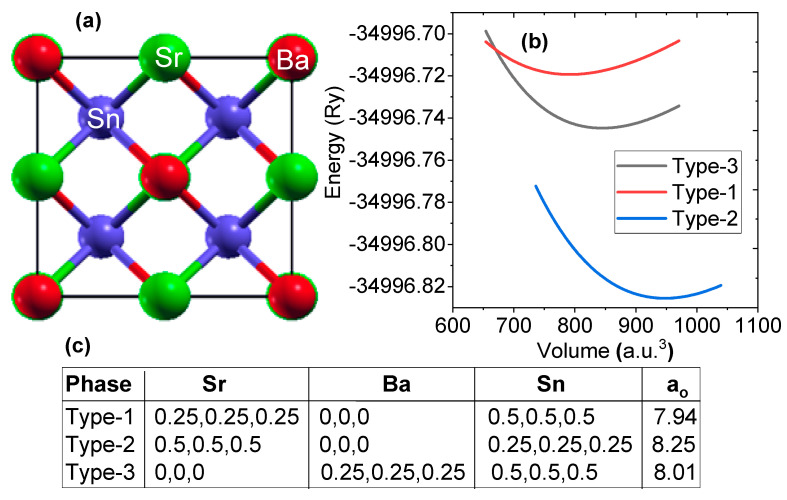
(**a**) Crystal structure of SrBaSn alloy in type-2 configuration. Red, green and blue colors represent Sr, Ba and Sn atoms, respectively, (**b**) energy versus volume curve and (**c**) possible atomic position of Sr, Ba and Sn atoms and lattice parameter (a_o_ in Å) of SrBaSn Heusler structure within F$\bar{4}$3 m space group in three different structural arrangements.

To determine the possible ground state atomic arrangements of SrBaSn, we optimized the three possible structural arrangements as listed in Figure 1. The site preferences of Sr, Ba and Sn atoms directly affect the electronic structure as well as other related properties of such half Heusler compounds. Energy–volume curve of SrBaSn within the above configurations via Murnaghan’s fitting [32] is displayed in Figure 1 to obtain the ground state lattice parameters as mentioned. From this data set, it is clear that the type-2 arrangement with energy minimum is the optimized configuration and the corresponding lattice constant (a_o_) is 8.25 Å. In the corresponding sections, we further check its elastic stability via stress–strain relations and thermodynamic stability via formation energy calculations. Additionally, the uniform application of pressure in both negative and positive directions is evaluated to check the phonon stability at equilibrium lattice parameter as well as against pressure. Since the formation energy calculations predicted previously find this material to be stable [4], we had confidence in studying its other electronic properties which can be thought of for its experimental synthesis.

### 3.2. Elastic Constants

Elastic constants in Table 1 computed for the SrBaSn alloy in its type-2-type ground-state stable structure include (*C*_11_, *C*_12,_ and *C*_44_), shear (G)/bulk (B)/Young (Y)) moduli, Poisson’s (ν), Pugh ratio (B/G), etc. Stability criterion proposed by Born–Huang [33] presumes the mechanical stability of SrBaSn in its Heusler structure form and is written in the form

(1) $$C_{11}\;>\;0,\;C_{44}\;>\;0,\;C_{11}\;-\;C_{12}\;>\;0\;\mathrm{and}\;C_{11}+2\;C_{12}\;>\;0$$

Later, the different moduli (B and V) are determined from the Voigt–Reuss–Hill approximations (VRH) [34,35], where B, G, Y, longitudinal ($v_{l}$) and transverse $\left(v_{t}\right)$ velocities are written as [36]

(2) $$B=\frac{\left(C_{11}+2C_{12}\right)}{3}$$

(3) $$G=\frac{\frac{\left(C_{11}-C_{12}+C_{13}\right)}{5}+\frac{5(C_{11}-C_{12})}{3\left(C_{11}-C_{12}\right)+4C_{44}}}{2}$$

(4) $$Y=\frac{9BG}{3B+G}$$

(5) $$v_{l}=\sqrt{\frac{G}{\rho }}$$

(6) $$v_{t}=\sqrt{\frac{\left(3B+4G\right)}{3\rho }}$$

Debye temperature $\theta_{D}$ via Anderson’s formula [37] in relation to $v_{l}$ and $v_{t}$ is determined below:

(7) $$\theta_{D}=\frac{\hbar }{k_{B}}{\left(\frac{3n\rho N_{A}}{4\pi M}\right)}^{1/3}[\frac{1}{3}\left(\frac{1}{v_{l}^{3}}+\frac{1}{v_{t}^{3}}\right){]}^{-1/3}$$

Here, the symbols have their usual meanings: (B/G) establishes the brittleness or ductility of a crystalline alloy [38] and B/G < 1.75 implies brittleness and vice versa. In the present case, the B/G value is 0.78, so SrBaSn is ductile in nature. Identical result is maintained by the negative Cauchy pressure (*C*_12_ − *C*_44_) value of 3.89, which characterizes its ductile nature and the positive value of (*C*_12_ − *C*_44_) predicts the brittle nature of the crystal [39,40]. Smaller Poisson’s ratio, e.g., 0.1–0.25, decides the easier fracture of a crystal and a larger Poisson’s ratio up to 0.5 means the hardness of a material to fissure. Additionally, negative poisons ratio is linked to non-central bonding forces (mostly observed in polymers and 2D materials), where the bonds are not strong enough and hence cracking occurs more easily as compared to the bulk crystals. An understanding was originally made by S.D. Poisson that if the atoms are interacting by central forces, and the deformation under a local strain combined with a rotation is applied, then Poisson’s ratio is 0.25 [41,42]. Additionally, when the computed Poisson’s ratio is 0.28 this defines the occurrence of central forces, its limiting value is 0.25, below which non-central bonds are predicted and the values between the range 0.25 and 0.50 depict the existence of central forces.

### 3.3. Phonon Stability

**Figure 2:**
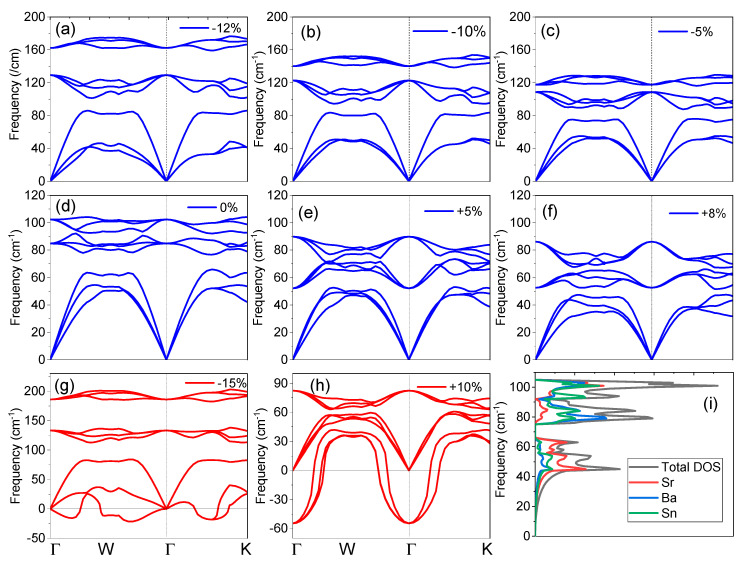
Phonon dispersion of SrBaSn at equilibrium and after strain application. (**a**–**c**) Compressive strain; (**d**) at equilibrium ground state (**e**,**f**) expansive strain; (**g**,**h**) represent unstable structures; (**i**) total and partial phonon density of states from each atom.

The vibrational frequencies are significant in view of dynamical and thermal properties of the SrBaSn crystal and hence the phonon dispersion curve without strain in the direction of (Γ-X-W-K-Γ) high symmetry K-points along with the total/partial density of the phonon states (pDOS) are displayed in Figure 2d,i. It is well established that no negative phonon modes are present and hence SrBaSn is dynamically stable. The three atoms in the SrBaSn unit cell produce nine phonon modes, viz: three acoustic and nine optical modes. The maximum of longitudinal acoustical phonon mode frequency at equilibrium is ~65 cm^−1^ and the lowest longitudinal optical phonon mode stabilizes at ~80 cm^−1^. The phonon band structure splits into three regions: a low frequency region (comprising three acoustic branches) from ~0–80 cm^−1^, mid frequency range (comprising three optical branches) from ~80–90 cm^−1^ and the high frequency region (comprising three optical branches) from ~90–105 cm^−1^.

Figure 2i illustrates the total and partial density of states (PDOS) of SrBaSn, which shows that the phononic states in the optic region are mainly contributed by the vibrations of Ba and Sn atoms while the major peak in PDOS in acoustical region is the mainly due to the contribution of Sr and Sn atoms. There is a clear separation of acoustic and optical phonon branches by 15 cm^−1^ and the two sets of optical branches (three each) are also separated. With the application of compressive strain up to −12% (see Figure 2a), these three branches get separated further till the negative frequencies or instability is observed at −15% strain (see Figure 2g). This results from the overlapping of electronic clouds beyond the extent of covalent radius of each atom and hence, repulsive forces become stronger and make the crystal instable. However, the transverse optical (TO) phonon modes are nearly flat along the symmetry directions during the course of strain application. In contrast, the expansive strain resists the stability till +10% only (see Figure 2h) as the bonding force (electron cloud overlapping) between the atoms is weakened and thus negative frequencies are observed.

### 3.4. Electronic Structure

**Figure 3:**
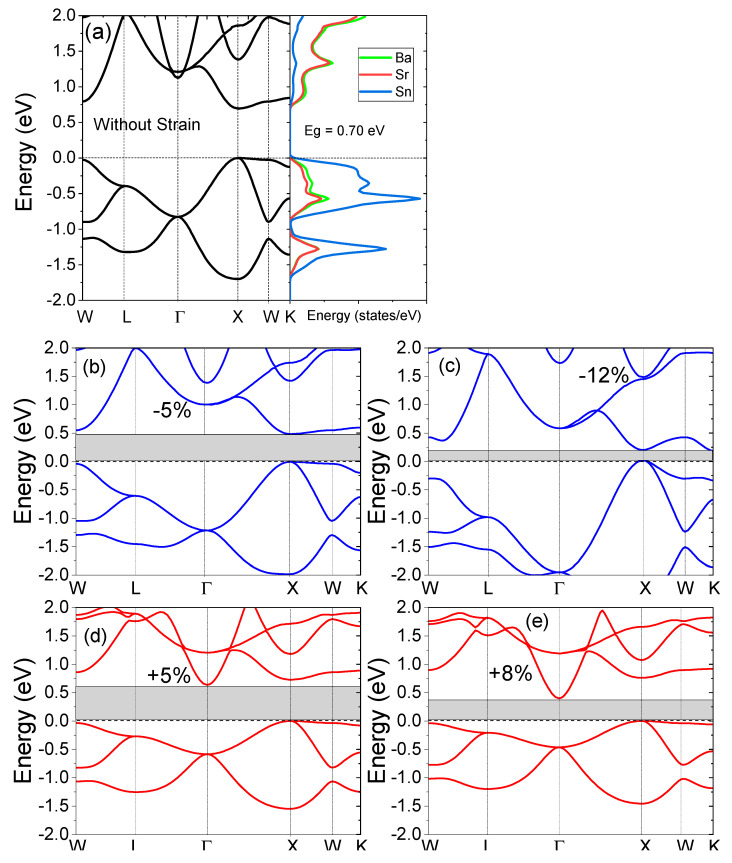
(**a**) Electronic structure and projected density of states (pDOS) with zero strain, (**b**) −5%, (**c**) −12%, (**d**) +5% and (**e**) +8% strain applied to the electronic structures of the SrBaSn material.

The electronic structure of the SrBaSn alloy and the density of the states are presented in Figure 3a. Since the valence electron count of this material is eight, its non-magnetic and insulating character is expected from the Slater–Pauling rule [43], stated as $M_{t}=(Z_{t}-18)\mu_{B}$. Here, $M_{t}$ is the total magnetic moment and $Z_{t}$ is the total number of valance electrons in its unit cell. So, the observed $M_{t}=0$ reflects the non-magnetic character of SrBaSn. In Figure 2a, the structure presents the minima of the conduction band (CBM) and the maxima of the valence band (VBM) to lie at X symmetry point, hence an indirect bandgap of 0.70 eV is observed at equilibrium lattice parameter. Additionally, the projected density of states (pDOS) in Figure 2b realizes the individual atomic orbital contribution with prominent Sn-p and Ba-s states hybridizing near the fermi level whereas the Sr-s states are lying low at the bottom of the valence band. At the same time, the empty conduction states are occupied mainly by Sr-s and Ba-s antibonding molecular orbitals. Our results are in accordance with the previous reports by Zhang et al. [4]. Moreover, the uniform strain application of +8% makes the CBM move 0.39 eV at Γ point and 0.75 eV at X point of the symmetry towards the fermi level. Hence, an indirect gap is observed after expansion. However, the −12% strain retains the direct nature of bandgap with CBM to shift 0.19 eV at X point and thus an overall decrease in bandgap is observed, while applying uniform strain. This behavior is due to a transition-metal-less structure where hybridization rather than exchange-correlation plays a vital role within the absence of d-d hybridization between d-less Sr and Ba-atoms. This feature is an important component in the formation of hollows in the density of states. The fate of such hollows to become actual bandgaps is strictly decided by the electronic structure details, which also include the relative size of the atoms and the relative positions of the atomic levels.

## 4. Conclusions

We investigated the structural, electronic and phonon dynamics of SrBaSn within the framework of density functional theory. At equilibrium lattice parameter, this material is found to be a direct bandgap semiconducting alloy with a gap of 0.70 eV. The ductile character from elastic parameters confirms the mechanical stability as well as the presence of central bonding forces. Strain application up to −12% and +8% sustains its phonon stability and beyond this the negative frequencies emerge as a consequence of instability.

## Figures and Tables

**Table 1:** Computed elastic parameters of SrBaSn compound at equilibrium lattice constant.

Parameter	Value	Parameter	Value
C_11_ (GPa)	49.16	$v_{l}$ (ms^−1^)	3275
C_12_ (GPa)	14.60	$v_{t}$ (ms^−1^)	2539
C_44_ (GPa)	10.71	$\theta_{D}$ (K)	163.98
B (GPa)	26.12	Pugh’s ratio (B/G)	0.78
G (GPa)	33.42	Poisson ratio (ν)	0.28
Y (GPa)	12.98	*C*_12_ − *C*_44_	3.89

## Data Availability

Not available.

## Sample Availability

Not available.
